# Spatial Distribution of Sediment Pesticides Concentrations in Streams of the Maritime Region of Canada

**DOI:** 10.1007/s00128-026-04241-y

**Published:** 2026-04-29

**Authors:** Benoit A. Lalonde

**Affiliations:** Environment and Climate Change Canada, Dartmouth (Nova Scotia), Canada

**Keywords:** Sediment, Pesticide, Streams, Persistence

## Abstract

This study measured pesticide concentrations at 51 sites in streams in three maritime provinces of Canada in 2020–2021. There were 50 pesticides analysed in each sediment samples. Most of those pesticides had concentrations below the detection limit of the laboratory. However, nine pesticides had detections that ranged from one to nine sites. Terbufos and parathion were the two most detected pesticides of this study. Surprisingly, terbufos has not been registered for use in Canada since 2012 which showcases the prevalence of some legacy contamination in sediments. Furthermore, the concentrations of terbufos, chlorpyrifos and clothianidin exceeded the calculated sediment threshold which indicates the potential for ecological harm. The results align with other recent research, suggesting that pesticide occurrence in sediment is complex and not explained by a single factor like organic matter or chemical properties alone. Instead, it is a result of multiple interacting factors, including land use, the pesticide’s solubility, its concentration in the surrounding water, and the persistence of legacy products.

**Supplementary Information:**

The online version contains supplementary material available at 10.1007/s00128-026-04241-y.

## Introduction

The Maritime region of Canada encompasses diverse agricultural zones shaped by soil composition, climate, and topography. Prince Edward Island and parts of New Brunswick, such as the Saint John River Valley, are particularly well-suited for potato cultivation due to their fertile, well-drained soils and temperate climate (Xing et al. 2012). Nova Scotia’s Annapolis Valley, benefiting from a unique microclimate, supports fruit production including apples, berries, and increasingly, wine grapes. Crop selection directly influences pesticide application intensity: orchard operations require frequent fungicide and insecticide treatments (Ernst 2009), while potato farming involves multiple applications of seed treatments, herbicides, and fungicides—often exceeding a dozen per season (AAFC 2022). In contrast, field corn typically requires fewer interventions, targeting early-season weed competition and specific pests (Ernst 2009). Pesticides enter aquatic ecosystems via surface runoff, erosion, and spray drift (Vryzas 2018), affecting water bodies ranging from small streams to major rivers like the Wolastoq (Saint John) River. Although surface water contamination has been documented (Lalonde & Garron 2020), sediment pollution remains underexplored.

Sediments are critical components of aquatic ecosystems and serve as long-term reservoirs for hydrophobic pesticides, which readily adsorb to organic matter and mineral surfaces (U.S. Geological Survey 2000). These compounds can re-enter the water column during high-flow events or through bioturbation, posing chronic risks to benthic invertebrates that form the base of aquatic food webs (Eggleton and Thomas 2004; Hunt et al. 2017; Nowell et al. 2016). Hydrophobic pesticides tend to resist desorption, remaining bound to sediment and creating persistent in-situ risks (Mahler et al. 2020). The potential for an inverse relationship between pesticide detection in water versus sediment—where less soluble compounds are missed in water samples but concentrated in sediment—underscores the need for sediment-focused analysis (Toth et al. 2024). Despite their ecological importance, sediment contamination data are scarce in Atlantic Canada and other regions such as the United Kingdom (Ramage et al. 2025). This study aims to address this gap by evaluating the detection rates and spatial distribution of pesticide residues in stream sediments across agricultural zones in the Maritime region.

## Materials and Methods

**Fig. 1:**
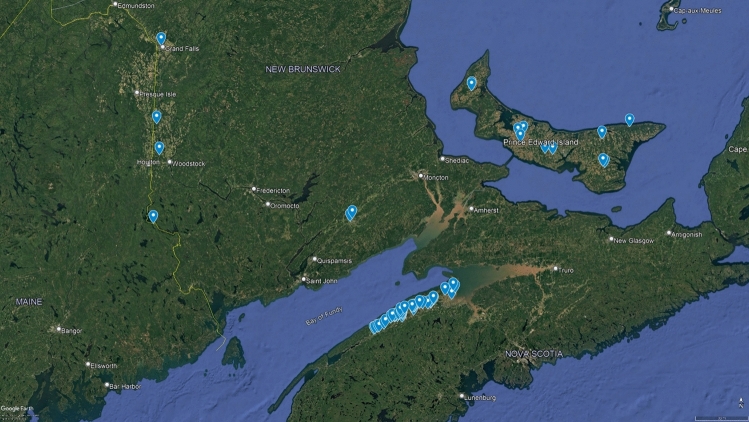
Map of the sampling sites (blue drops) located in the Maritime Provinces of Canada

A total of 51 sampling sites were distributed across agricultural regions in the Maritime provinces of Canada, with the majority located in Nova Scotia and Prince Edward Island, and only six sites in New Brunswick (Fig. 1). Site metadata including coordinates and stream order (ranging from 1–4) are detailed in Supplementary Table 1. Sediment samples for pesticide and grain size analysis were collected using laboratory-certified 500 mL amber glass jars with Teflon caps. Depending on watercourse size and depth, surface sediments (top 3 cm) were retrieved using either a stainless steel spoon or an Eckman dredge. Samples were handled with clean polyethylene gloves, stored in the dark, refrigerated, and delivered overnight to ECCC’s Atlantic Laboratory Environmental Testing (ALET) in Moncton, NB. Samples were frozen at − 20 °C, thawed prior to preparation, and extracted within 12 months of collection. Final extracts were stored at <  − 10 °C and analyzed within 12 months of extraction (ALET 2024).

Particle size analysis was conducted at ECCC’s Prairie and Northern Laboratory using Method 180.4, involving drying, sieving, and laser diffraction via the Horiba Partica LA-950V2 (ECCC 2024). Both ECCC laboratories used in this study are both ISO 17025 and CALA certified. Pesticide quantification was performed at ALET using QuEChERS-based extraction and cleanup, followed by either gas chromatography–mass spectrometry (GC-MSMS) or liquid chromatography–mass spectrometry (LC-MSMS), depending on compound characteristics (ALET 2024). Detection limits ranged from 1.45–2.9 ng/g dry weight, based on a 3 g sample and 1 mL final extract volume. GC-MSMS utilized a customized Agilent system with hot ACN:MES injection and MRM detection, while LC-MSMS employed a Waters Xevo TQS-micro system with FTN injection and compound-specific MRM transitions. A 10-point calibration curve was used for quantification, with correction factors applied for solid matrices. Quality control included procedural blanks, spikes, and calibration checks at low, mid, and high concentrations (ALET 2024).

## Results and Discussion

Fifty pesticides were monitored during the study, but quantifiable concentrations were not found for 41 of them, including commonly used compounds such as atrazine, imidacloprid, and malathion. Nine pesticides were detected in sediment samples across Nova Scotia and Prince Edward Island, with varying frequencies and environmental relevance (Table 1). Only nine pesticides were detected, with terbufos and parathion being the most frequently observed, found at nine and eight sites respectively. Notably, no detections occurred at any of the six New Brunswick sites, while the Dunk River (PEI) site had the highest number of pesticide detections (Table 1). Environmental fate characteristics (Kow, solubility, etc.) of the detected pesticides are listed in supplementary information Table 13.

**Table 1:** Detection frequency, average concentration detected and province where pesticides were measured during this study

Pesticide	# Detections	Range (ng/g)	Average concentration (ng/g)	Avg detection limit (ng/g)	Province
Trifluralin	1	2.01	2.01	1.97	PEI
Linuron	2	1.5–2.0	1.7	1.7	NS, PEI
Chlorpyrifos	2	2.1–3.4	2.73	1.99	NS, PEI
Metolachlor	2	3.4–9.9	6.65	2.5	NS
Clothianidin	2	1.9–2.2	2.12	1.98	PEI
Pendimethalin	4	2.3–16.1	9.41	2.16	NS
Chlorantraniliprole	5	3.5–9.2	5.13	2.2	NS, PEI
Parathion	8	1.5–2.9	2.49	1.99	NS, PEI
Terbufos	9	2.3–5.8	3.69	2.41	NS

Pendimethalin, known for its strong soil binding (EPA 1997), was found at four sites in Nova Scotia, particularly where silt content was highest. Chlorantraniliprole, moderately persistent and prone to sediment partitioning (HC 2014), was detected at seven sites, mostly in PEI, aligning with previous water sampling data (Lalonde and Garron 2020). Chlorpyrifos, banned in Canada since 2023 (HC 2019), was detected at low concentrations in two PEI sites, contrasting with its absence in Québec sediments (Toth et al. 2024). Trifluralin, with low solubility and high soil affinity (HC 1992), was detected at only one site, consistent with its low water detection rate in the region (Lalonde and Garron 2020). Parathion, the second most frequently detected pesticide (16%), was absent from water samples but present in sediments across a wide range of silt concentrations, suggesting no clear sediment affinity (Richardson 2005; Lalonde and Garron 2020). Terbufos, banned in Canada since 2012 (PMRA 2003), was the most frequently detected pesticide in this study, likely due to its persistence and sediment-binding properties (HC 1995; Mamo et al. 2006). Clothianidin, despite its high detection in water (90%; Lalonde and Garron 2020), was found in only 4% of sediment samples, likely due to its high solubility and low partitioning (EPA 2007; HC 2018), consistent with findings from Main et al. (2014) and contrasting with higher detection rates in China (Zhang et al. 2025). Linuron and metolachlor were each detected in 4% of sediment samples, consistent with their low water detection rates in the region (Lalonde & Garron 2020). Linuron showed no consistent relationship with silt content, possibly reflecting localized crop use (PMRA 2020), while metolachlor’s lower sediment detection compared to Québec (Toth et al. 2024) may be due to regional crop differences and its persistence in aquatic environments (EXTOXNET 2019).

Grain size analysis revealed seven sampling sites with silt content ≥ 30%, with Valleyfield (PEI) exhibiting the highest proportion at 70.8%. While previous studies have shown that pesticides tend to associate with finer sediment fractions such as silt (Vryzas 2018; Toth et al. 2024), the relationship in this study was inconsistent. Sites like Dunk and Wilmot, which had elevated silt levels, also showed high pesticide detection frequencies, yet Valleyfield and Knox—both with > 40% silt—had minimal or no detections, suggesting that silt content alone may not reliably predict pesticide occurrence. Toth et al. (2024) further explored the role of hydrophobicity, measured via the n-octanol-water partition coefficient (Kow), in sediment binding. Although pesticides such as chlorpyrifos, metolachlor, and linuron possess high Kow values (> 3), their detection frequencies in sediment were low, while lower Kow compounds like atrazine and carbaryl were more frequently detected (46 and 37%, respectively, Toth et al. 2024). These findings suggest that pesticide presence in sediment may be more strongly influenced by water column concentrations and desorption dynamics than by sediment affinity alone.

Although no prior studies on pesticide concentrations in freshwater sediments were identified for the Maritime region of Canada, comparisons with research conducted elsewhere offer valuable context (Table 2). Giroux (2023) reported detections for only 18 of 77 pesticides in Québec sediments, with glyphosate and AMPA being most frequent (78 and 8%, respectively), though these were not analyzed in the present study. Chlorantraniliprole, the third most detected pesticide in Giroux (2023), had a detection rate of 15%, comparable to the 10% observed here (Table 2). Their study found a statistically significant correlation between pesticide detections and the proportion of vegetable-growing land, while organic matter showed no significant relationship. In contrast, Ramage et al. (2025) found total organic carbon to significantly influence pesticide concentrations in UK sediments (*p* < 0.01, R^2^ = 0.645), highlighting the complexity of sediment-pesticide interactions. Toth et al. (2024) also observed widespread pesticide detections in Québec sediments (47% of 30 pesticides) but found no correlation between sediment binding and high Kow values, suggesting that pesticide occurrence is governed by multiple interacting factors. International comparisons further support the role of legacy contamination: Wei et al. (2021) reported higher terbufos concentrations in Chinese sediments, while Prajapati et al. (2022) detected banned pesticides such as methoxychlor and lindane decades after their prohibition in Canada. Similarly, Knight et al. (2007) found pp’-DDT and dieldrin in U.S. sediments long after their last legal use. Parathion, detected in 16% of sites in this study, was found at higher concentrations than those reported by Prajapati et al. (2022) in Saskatchewan (3%), possibly reflecting regional differences in crop use. A review on the occurrence of organic contaminants in Europe by Ducrocq et al (2024) based on 163 peer reviewed articles revealed detections of the nine pesticides of this study but also the detections of over 100 other pesticides not detected in this study.

**Table 2:** Range of pesticide concentrations, detection frequency in sediment from other studies

Pesticide	Range (ng/g)	Detection frequency (%)	Locations	Study
Chlorantraniliprole	ND-102	1	Canada	Toth et al (2024)
Metolachlor	ND-1237	34	Canada	Toth et al (2024)
Chlorantraniliprole	0.5–9.2	15	Canada	Giroux et al. (2023)
Clothianidin	0.5–24	6.1	Canada	Giroux et al. (2023)
Clothianidin	2.6–4.4	6.0	Canada	Main et al (2014)
Parathion	0–0.91	3.7	Canada	Prajapati et al (2022)
Clothianidin	ND-1.91	61–100	China	Zhang et al. (2019)
Chlorpyrifos	Nd-6.31	27.6–37.9	China	Wei et al. (2021)
Terbufos	ND-30.6	20.7–41.4	China	Wei et al (2021)
Clothianidin	< 0.2–11.93	31–55	United States	Kuechle et al. (2019)
Metolachlor	ND-107.1	69	United States	Knight et al (2007)
Trifluralin	ND-1.2	19	United States	Knight et al (2007)
Pendimethalin	ND-39.4	11	United States	Knight et al (2007)
Chlorpyrifos	ND-34.3	33	United States	Knight et al (2007)
Chlorpyrifos	ND-440	n/a	United States	Smalling et al (2012)
Pendimethalin	ND-13.2	n/a	United States	Smalling et al (2012)
Clothianidin	ND-1.22	26–30	United Kingdom	Ramage et al. (2025)

ND: Non-detect

The findings of this study reveal that pesticide occurrence in sediment is not consistently explained by silt content or chemical properties such as the n-octanol-water partition coefficient (Kow), despite their theoretical relevance. While silt may serve as a general indicator of potential pesticide presence (Vryzas 2018; Toth et al. 2024), its predictive power was limited across sites in the Maritime region of Canada. Similarly, high Kow values did not correlate with detection frequencies, underscoring the complexity of sediment-pesticide interactions.

Currently, none of the pesticides identified in this study are subject to established Canadian federal sediment quality guidelines. To assess potential ecological risk, sediment threshold values were derived using the Equilibrium Partitioning method (EqP). This approach integrated chronic water quality guidelines from the Canadian Council of the Ministers of the Environment or the United States Environmental Protection Agency chronic benthic invertebrate benchmarks with chemical-specific organic carbon partition coefficients (Koc) and a predicted total organic carbon (TOC) concentration of 3% (Table 3). The formula for the Eqp is EqP = Water quality guidelines * Koc * %TOC. The TOC value was selected as a very conservative baseline for agricultural stream sediments within the Maritime region. In addition, risk quotients were calculated using the average sediment concentrations measured in this study and the calculated threshold values (Table 3). Analytical results indicate that concentrations chlorpyrifos, clothianidin and terbufos exceeded these calculated thresholds suggesting a high probability of adverse toxicological impacts on benthic invertebrate communities.

**Table 3:** Water quality guidelines, KOC, calculated thresholds and risk quotients for detected pesticides in Maritime Rivers of Canada

Pesticides	WQG* (µg/L)	Koc** (L/kg)	Calculated threshold (ng/g)	Risk quotient	*WQG references
Chlorpyrifos	0.002	5509	0.33	8.27	CCME (2026)
Clothianidin	0.05	123	1.12	1.89	EPA (2026)
Terbufos	0.03	500	0.45	1.4	EPA (2026)
Parathion	0.013	7660	2.98	0.84	EPA (1986)
Metolachlor	7.8	120	28.1	0.24	CCME (2026)
Chlorantraniliprole	3.02	362	32.79	0.15	EPA (2026)
Trifluralin	0.2	15,800	94.8	0.02	CCME (2026)
Linuron	7	842.8	176.9	0.012	CCME (2026)
Pendimethalin	14.5	17,491	7608	0.001	EPA (2026)

** Koc values from 2026. University of Hertfordshire. PPDB: Pesticide properties database. Access; Pesticide properties database

These results emphasize the importance of sediment monitoring alongside surface water sampling to identify environmental risks that might otherwise go undetected. Moreover, the presence of banned pesticides such as terbufos and parathion suggests ongoing legacy contamination. Overall, pesticide occurrence in sediment appears to be governed by multiple interacting factors, including land use, solubility, lipophilicity, and persistence, rather than any single variable.

## Supplementary Information

Below is the link to the electronic supplementary material.

Supplementary file1 (DOCX 30 kb)
