# Supplementary material for: Spatial Distribution of Sediment Pesticides Concentrations in Streams of the Maritime Region of Canada
Source: Bull Environ Contam Toxicol. 2026 Apr 29;116(5):100. doi: 10.1007/s00128-026-04241-y (PMC13124949; doi:10.1007/s00128-026-04241-y)
Supplement: Supplementary file 1 — Supplementary file1 (DOCX 30 kb) [file 128_2026_4241_MOESM1_ESM.docx]

Table 1. Sampling Sites characteristics

| Year | Site | Coordinates (NAD 83) | Stream Order |  | Year | Site | Coordinates (NAD 83) | Order |
| --- | --- | --- | --- | --- | --- | --- | --- | --- |
| 2021 | Black Brook | N44° 57' 05.8" W65° 00' 01.2" | 3 |  | 2020 | Sharpe | N45° 03' 28" W64° 38' 02" | 3 |
| 2021 | Leonard | N44° 52' 13.7" W65° 12' 00.5" | 2 |  | 2020 | Coleman | N45° 05' 04" W64° 35' 56" | 3 |
| 2021 | Lily Brook | N44° 56' 27.1" W65° 04' 27.8" | 2 |  | 2020 | Lawrence | N45° 05' 23" W64° 36' 21" | 2 |
| 2021 | Morton Brook | N44° 57' 06.2" W65° 03' 19.7" | 2 |  | 2020 | Little | N44° 58' 45" W64° 57' 54" | 2 |
| 2021 | Munroes | N44° 51' 18.1" W65° 14' 44.6" | 2 |  | 2020 | Armstrong | N45° 00' 44" W64° 55' 17" | 2 |
| 2021 | Saunders | N44° 51' 06.9" W65° 15' 33.8" | 1 |  | 2020 | Graves | N45° 01' 30" W64° 50' 22" | 2 |
| 2021 | Shearer | N44° 52' 05.4" W65° 13' 07.5" | 2 |  | 2020 | Patterson | N45° 01' 41" W64° 49' 20" | 2 |
| 2021 | Spinney | N44° 56' 51.3" W64° 56' 57.1" | 3 |  | 2020 | Walker | N44° 59' 52" W64° 58' 06" | 2 |
| 2021 | Zeke | N44° 58' 37.5" W64° 56' 33.7" | 3 |  | 2020 | Oak | N44° 53' 26" W65° 08' 50" | 2 |
| 2021 | Cornwallis | N45° 03' 53.7" W64° 38' 07.8" | 4 |  | 2020 | McEwan | N44° 54' 25" W65° 07' 50" | 3 |
| 2021 | Up Cornwallis | N45° 03' 11.6" W64° 43' 28.3" | 4 |  | 2020 | Wiswal | N44° 57' 57" W64° 59' 23" | 2 |
| 2021 | North | N45° 09' 31.3" W64° 28' 41.1" | 2 |  | 2020 | Annapolis | N44° 56' 57" W65° 01' 46" | 4 |
| 2021 | Barkhouse | N45° 10' 39.7" W64° 21' 59.3" | 1 |  | 2020 | Fales | N44° 57' 58" W64° 56' 33" | 3 |
| 2021 | Canard | N45° 07' 37.3" W64° 24' 18.8" | 3 |  | 2020 | Rand | N45° 03' 23" W64° 45' 31" | 2 |
| 2021 | Pereaux | N45° 11' 36.6" W64° 23' 17.7" | 2 |  | 2020 | South | N45° 00' 07" W64° 49' 21" | 3 |
| 2021 | Rochford | N45° 03' 07.1" W64° 40' 02.8" | 3 |  | 2020 | Skinner | N45° 01' 46" W64° 48' 45" | 2 |
| 2020 | Little River | N47° 03' 11.5" W67° 44' 19.7" | 3 |  | 2020 | Watton | N44° 57' 20" W65° 01' 36" | 2 |
| 2020 | Big Presqu’Ile | N46° 26' 20.9" W67° 44' 44.0" | 4 |  | 2020 | Valleyfied | N46° 08' 19" W62° 40' 39" | 3 |
| 2020 | Meduxnekeag | N46° 13' 39.3" W67° 44' 27.4" | 4 |  | 2020 | West | N46° 15' 20" W63° 21' 17" | 3 |
| 2020 | Kennebecasis | N45° 43' 28.4" W65° 32' 14.9" | 4 |  | 2020 | Morell | N46° 21' 47" W62° 42' 09" | 2 |
| 2020 | Smiths Creek | N45° 44' 52.6" W65° 30' 44.1" | 3 |  | 2020 | Bear | N46° 27' 12" W62° 22' 57" | 2 |
| 2020 | St Croix | N45° 39' 51.2" W67° 44' 00.0" | 4 |  | 2020 | Montague | N46° 08' 55" W62° 41' 55" | 3 |
| 2020 | Mill | N46° 44' 38.0" W64° 10' 53.6" | 2 |  | 2020 | Caines | N46° 23' 36" W63° 39' 29" | 2 |
| 2020 | Cains | N46° 45' 17.4" W64° 10' 50.4" | 3 |  | 2020 | Wilmot | N46° 24' 28" W63° 35' 48" | 3 |
| 2020 | Dunk | N46° 20' 46.1" W63° 37' 57.1" | 4 |  | 2020 | Clyde | N46° 14' 47" W63° 16' 06" | 3 |

Table 2. Environmental Fate Characteristics

|  | CAS # | Water Solubility (20c) (mg/L) | log P | Soil degradation (days) | Hydrolysis (days) | Water-sediment DT50 (days) | BCF (l/kg) | Log Kow |
| --- | --- | --- | --- | --- | --- | --- | --- | --- |
| Terbufos | 13071-79-9 | 4.5 | 4.5 | 8 | 6.5 | n/a | 286 | 4.48 |
| Parathion | 56-38-2 | 12.4 | 3.83 | 49 | 260 | 4.3 | 40 | 3.83 |
| Pendimethalin | 40487-42-1 | 0.33 | 5.4 | 182.3 | Stable | 16 | 5100 | 5.18 |
| Chlorantraniliprole | 610-489-8 | 0.88 | 2.86 | 597 | Stable | 170 | 15 | 2.76 |
| Clothianidin | 433-460-1 | 327 | 0.90 | 545 | Stable | 56.4 | 1.12 | 0.73 |
| Linuron | 330-55-2 | 63.8 | 3 | 57.6 | 1460 | 24.1 | 49 | 3.2 |
| Chlorpyrifos | 2921-88-2 | 1.05 | 4.7 | 386 | 53.6 | 36.5 | 1374 | 5 |
| Metolachlor | 51218-45-2 | 530 | 3.4 | 90 | Stable | 365 | 68.8 | 2.9 |
| Trifluralin | 1582-09-8 | 0.221 | 5.27 | 133.7 | stable | 5.5 | 5674 | 5.07 |
